# Magnetic Resonance Imaging in Multiple Sclerosis – Patients' Experiences, Information Interests and Responses to an Education Programme

**DOI:** 10.1371/journal.pone.0113252

**Published:** 2014-11-21

**Authors:** Judith Brand, Sascha Köpke, Jürgen Kasper, Anne Rahn, Imke Backhus, Jana Poettgen, Jan-Patrick Stellmann, Susanne Siemonsen, Christoph Heesen

**Affiliations:** 1 Institute of Neuroimmunology and Clinical MS Research (INIMS) and Dep. of Neurology, UMC Hamburg Eppendorf, Hamburg, Germany; 2 Institute for Social Medicine and Epidemiology, University of Lübeck, Lübeck, Germany; 3 Department of Primary Medical Care, UMC Hamburg Eppendorf, Hamburg, Germany; 4 Unit of Health Sciences and Education, MIN Faculty, University of Hamburg, Hamburg, Germany; 5 MS Imaging Section, Department of Diagnostic and Interventional Neuroradiology, UMC Hamburg Eppendorf, Hamburg, Germany; University Hospital Basel, Switzerland

## Abstract

***Background*:**

Magnetic resonance imaging (MRI) is a key diagnostic and monitoring tool in multiple sclerosis (MS) management. However, many scientific uncertainties, especially concerning correlates to impairment and prognosis remain. Little is known about MS patients' experiences, knowledge, attitudes, and unmet information needs concerning MRI.

***Methods*:**

We performed qualitative interviews (n = 5) and a survey (n = 104) with MS patients regarding MRI patient information, and basic MRI knowledge. Based on these findings an interactive training program of 2 hours was developed and piloted in n = 26 patients.

***Results*:**

Interview analyses showed that patients often feel lost in the MRI scanner and left alone with MRI results and images while 90% of patients in the survey expressed a high interest in MRI education. Knowledge on MRI issues was fair with some important knowledge gaps. Major information interests were relevance of lesions as well as the prognostic and diagnostic value of MRI results. The education program was highly appreciated and resulted in a substantial knowledge increase. Patients reported that, based on the program, they felt more competent to engage in encounters with their physicians.

***Conclusion*:**

This work strongly supports the further development of an evidence-based MRI education program for MS patients to enhance participation in health-care.

## Introduction

People with multiple sclerosis (MS) give information about magnetic resonance imaging (MRI) and about its relevance for diagnosis and prognosis highest priority [1]. Currently, MRI is the most important para-clinical tool in the diagnosis and management of MS, especially in monitoring treatment effects.

However, there are substantial scientific uncertainties in the application of MRI which need to be communicated to patients. Based on MRI, MS can be diagnosed now after a single clinical event [2] which means a very early confrontation of patients with a non curable possibly sub-clinical chronic disease. Using these more sensitive criteria, conversion rates to clinical definite MS might be lower than based on older criteria [3]. Diagnostic accuracy, i.e. sensitivity and specificity of MRI, however remains unsatisfactory [4]. A radiological isolated syndrome (RIS) has been defined as a pre-stage of MS without clinical signs solely based on MRI even more eliciting the question of conversion rates and treatment needs [5]. The number of lesions at first presentation as well as the increase in lesion load during the first 5 years of MS has shown some prognostic value, however these findings are based on a single cohort with 107 patients [6]. Short-term epidemiological studies and findings from MS treatment trials are inconclusive. Metaanalytic work from n = 223 patients in 31 placebo cohorts of MS treatment trials has for example shown that T2 lesion load and Gadolineum enhancement has no independent prognostic value for disability [7]. In another review from epidemiological and treatment studies (n = 302 patients) Gadolinium enhancement was not predictive of disability progression [8]. On the other hand recent review work of interferon-beta treatment trials postulate a predictive value of new T2 lesions and Gadolineum enhancement for relapse activity and disability progression when occurring on treatment [9]. Although persistent MRI activity during disease modifying drug (DMD) treatment is considered a criterion of non-response, no consensus has been obtained to judge responsiveness solely on an MRI base [10]. In the future, newer MRI techniques might improve the so far limited clinical correlates and prognostic value [11].

In clinical practice, the scientific uncertainties concerning MRI are not mirrored in patients' knowledge. Based on clinical experience, physicians tend to overemphasize the predictive value of MRI. Clinical experience indicates important divergence in usage of MRI. However, to our knowledge systematic care-oriented research data on how MRI is applied in daily life are missing. Consensus criteria on relevant MRI sequences in clinical management have been suggested [12], but monitoring frequency criteria only exist on a center basis [13]. In addition MRI is a costly medical procedure.

In Germany, patients tend to take home MRI images on CD as well as a radiological report, but no standards on disclosure of findings exist among radiologists, neuroradiologists, or neurologists. While on the one hand patients have access to their own images, on the other hand they report fear and lack of knowledge on how to interpret MRI images and reports. This is in contrast to numerous studies showing that MS patients aim for active roles in the management of their disease [14],[15]. In order to enable such a role, patients demand and need evidence-based information on the complex issue of MRI.

We performed a systematic literature search in PubMed on the topics MS, MRI, and patient education and patient information to clarify the current stage of research, which yielded 312 hits with no relevant studies identified after title and abstract screening (see suppl. data).

Therefore, we studied patients' experiences, knowledge and interest concerning MRI using qualitative and quantitative survey methodology. We hypothesized a substantial perceived threat concerning the investigation and important knowledge gaps. As a result an evidence-based patient education program on MRI in MS was developed and piloted as a group training. We assumed that carefully developed information not only increases knowledge but also motivates patients to engage more in medical decision making.

## Methods

This work is part of a larger study on patient information and coaching on immunotherapy decisions. The study on the development of a MRI education program has been specifically approved by the Ethics Committee of the Hamburg Chamber of physicians (number PV4576). For the survey, written informed consent was obtained from all participants. All participants of the pilot education program enrolled voluntarily.

### Qualitative Research

In a first step, five patients from the MS Outpatient Clinic of the University Medical Center (UMC) Hamburg Eppendorf, Germany, were recruited for semi-standardized interviews consisting of nine open questions regarding their experiences with MRI and their areas of interests as well as preferences for contents and structure of an MRI education program. The interviews were audio-recorded and analyzed and content analysis was guided by the thematic framework analysis of Ritchie and Spencer [16]. The aim of these interviews was to assess patients' perceptions and preferences concerning a questionnaire to be used in a representative survey.

### Survey

A 32-item questionnaire was developed on MRI issues and sent via email to 200 randomly selected MS patients from the database of the MS outpatient registry of the UMC Hamburg Eppendorf, who had presented between 11/2010 and 11/2012 (n = 1374). The sample size was based on previous survey results [1]. We included patients with either long-term disease duration (time since diagnosis ≥10 years) or short-term disease duration (time since diagnosis ≤5 years) following the hypothesis that patients with longer disease duration have more MRI experience and higher MRI knowledge scores than patients with a more recent diagnosis. Most patients of the outpatient clinic present once or twice a year especially when the disease is already established for some time.

Eight letters were returned undeliverable due to a change of address and contact details. The overall response rate of the questionnaire was 58% (112 out of 192). Out of 112 returned mails, 73% (n = 82) were filled-in, while the remaining 27% (n = 30) patients gave a feedback of not being interested (20 female and 10 male), of which 47% (14) gave ‘No interest in educational program’ as a reason. Other reasons were: ‘no time’, ‘no interest in MS’ and ‘no MS diagnosis’. 22 further consecutive patients from the MS Outpatient Clinic of the UMC Hamburg Eppendorf fulfilling the inclusion criteria were asked in November 2012 to complete the survey adding to 104 analyzable questionnaires.

### MRI questionnaire

The questionnaire contained four parts with a total of 32 items, 26 of which were newly developed within the research team. Four items were taken from an own MS risk knowledge questionnaire (RIKNO) [17], two derived from the Hamburg Quality of Life in MS questionnaire (HAQUAMS) [18]. The questionnaire covered the following topics:

Part 1 (6 items): MS demographic data and disease associated anxiety and depressive mood. On an ordinal scale patients indicated perceived distress during an MRI investigation.

Part 2 (9 items): Patients' experiences with MRI regarding frequency and communication about findings with their physician.

Part 3 (5 items): Patients' interests, ideas and preferences for a patient education program concerning length, group size, and MRI topics. In order to specify the fields of interest within an MRI education module, different topics were presented (1 item). Other items addressed the preferred format of the education program (4 items).

Part 4 (12 items): MRI knowledge assessment (see appendix) comprised 11 multiple-choice questions that were summarized to an MRI knowledge score of 17 possible points (see appendix). Questionnaire items addressed neuro-anatomy (1 item, 7 points), practical issues of MRI conduct (3 items, 3 points), basic knowledge on brain lesions (2 items, 2 points) and the value of the MRI for diagnosis and prognosis as well as DMD treatment effects (5 items, 5 points). Subjective MRI knowledge was assessed using a visual analogue scale from ‘no knowledge’ to ‘highest knowledge’ as applied earlier [1]. The scale was divided into 10 sections with 10 representing highest knowledge (1 item).

### Development and evaluation of the education program

Based on the results of the qualitative study and the survey, a power point-based education program was developed, covering the most relevant information on MRI for MS patients. The initial draft was discussed and revised several times in our work group (JB, CH, JK, SK, SS). Corresponding to the concept of evidence-based patient information [19], contents were based on literature researches and two systematic reviews, one concerning ‘MRI and diagnosis’ [20] and another concerning ‘MRI and prognosis’ (manuscript under preparation).

The electronic patient newsletter of the MS Day Hospital was used to recruit participants for a pilot training session. The program was presented by JB, an MS educated medical student to 26 MS patients who responded to the newsletter in two pilot groups of 13 patients each. After the 90-minute presentation, an open question and answer round was conducted. Participants' comments were audio-recorded and analyzed using content analysis [16].

Patients' knowledge on MRI was tested using a questionnaire with 15 knowledge questions based on the survey. It was administered directly before and directly after the education program. The quality of the program was assessed using 4-point Likert scales, where patients marked the level of agreement to given statements. Three domains of quality were evaluated: satisfaction with the education program (9 items), anticipated effects of the increased MRI knowledge (7 items), and the assumed impact on patient-physician communication (6 items). Mean item scores of the three domains were summarized to three sub-scores.

### Ethical issues

This work is part of a larger study on patient information and coaching on immunotherapy decisions and has been agreed upon by the Ethics Committee of the Hamburg Chamber of physicians (number PV4576). Informed consent was obtained from all participants.

### Statistical analysis

Most data were analyzed descriptively using SPSS 21.0 for Windows. We performed t-tests for independent samples to analyze MRI knowledge score differences between the two patient groups in the first survey. Correlation between subjective and objective knowledge in the survey was analyzed using Fisher's-Z-test in order to generate Pearson correlation coefficients. T-tests for paired samples in the evaluation of the program were conducted to assess before-after comparisons of subjective and objective knowledge.

## Results

### Qualitative study (Table 1)

The patient group consisted of five female patients with relapsing-remitting MS (RRMS) aged between 22 and 48 years with an average disease duration of four years.

**Table 1:** Interview and focus group findings.

MRI experience		
*Major category*	subcategory	Patient statement
*To be at the mercy of the investigation*	Noisiness and narrowness	“It was pretty loud and narrow. The narrowness is a problem.”
	Relaxation strategies	“Other patients recommended MRI practices where I might pick my favorite music.”
*To be at the mercy of the results*	Incomprehension	“The doctor reviewed the images with me, but I did not understand what he was saying.”
	Information timing	“Only the diagnosis M” was of importance for me. I did not care about images. I felt like being in a movie, everything just passed by.”
	Non-disclosure of findings	“Images were neither shown nor explained to me, just handed out in an envelope.”
	Disgust	“Seeing the inner body feels a bit disgusting, especially the eyes.”
**Expectations towards MRI education**		
*Self-management of MRI images*	Understanding images	“I felt better once I had received the diagnosis. I want to know where the wind blows.”
	Understanding reports	“Being able to read and understand the doctor's report would be great.”
	Own comparisons	“Being able to compare the images myself and understand what the doctors really talk about.”
	Being independent from physician	“To know about my own body and not having to rely on the doctor all the time.”
	Empowerment within physician encounters	“I would like to be prepared better for medical consultations.”
*Ambivalence of a deeper understanding*	Interest in neuro-anatomy	“I would like to know more about different areas of the brain function.”
	Clinical correlate of images	“I can see a white spot. That means there was a relapse.”
	Excitement towards results	“I find it fascinating even though I fear my results.”
	Fear of unfavorable prognostic information	“My only concern would be the MRI showing me the future of my disease. The other question is, if this is really possible?”

All five interviewees showed considerable interest in MRI, mostly reporting a substantial lack of knowledge and considerable fear, not only concerning the results, but also concerning the procedure itself. For the stage of the diagnostic process participants reported ambivalence towards having a deeper insight into MRI issues. In contrast, all interviewees stressed the need for more insight into MRI issues during the further course of the disease. In general, MRI was perceived as a procedure where patients felt substantially uncomfortable, not only during the procedure itself but also while receiving information on the results. A putative deeper insight through an education program was associated with ambivalence as some interviewees feared the disclosure of a potentially unfavorable prognosis.

Asking patients to assess a T1 weighted coronal planed image on the level of the eyes led to excitement and interest in four patients and disgust in one patient.

### Survey

104 questionnaires were analyzed. Participants had an average age of 48 years (range: 19–69). 43 had short disease durations of ≤5 years, while 61 had disease durations of ≥10 years. As expected, there were more participants with progressive disease courses in the patient group with longer disease durations (table 2).

**Table 2:** Demographic data of survey on MRI experiences.

Time since diagnosis	0–5 years	>10 years	all
n (%)	43 (41.3)	61 (58.7)	104 (100)
Disease duration, years (mean ±SD)	1.23 (1.65)	19.80 (8.59)	11.3 (11.9)
RRMS	27 (62.8)	9 (14.8)	36 (34.6)
PPMS	1 (2.3)	6 (9.8)	7 (6.7)
SPMS	4 (9.3)	38 (62.3)	42 (40.4)
Disease course unclear	11 (25.6)	8 (13.1)	19 (18.3)
Ongoing immunotherapy	13 (30.2)	19 (31.1)	32 (30.8)
High level of education*	25 (58.1)	40 (65.6)	65 (62.5)
Subjective MRI knowledge** (mean, SD)	4.27 (2.17)	4.49 (2.33)	4.42 (2.27)
MRI knowledge*** (mean, SD)	10.51 (3.18)	9.57 (3.39)	9.96 (3.32)

Values are numbers (%) if not indicated differently.

* 12 or more years of school,

** Range 0–10 with higher values indicating good knowledge.

*** Objective MRI knowledge (range 0–17 with higher values indicating good knowledge).

### MRI usage

While 43.3% (n = 45) indicated irregular MRI scans, 17.3% (n = 18) reported a frequency of one MRI per year, followed by 10.6% (n = 11) with two MRI per year. Only 2 out of 104 patients had more than 2 MRI per year. Of patients with longstanding MS, 56.7% tended to have irregular MRI opposed to 20.6% of patients with a more recent diagnosis. 26.5% only had one MRI during the last 2 years. 76.5% reported to have repeated images performed at the same scanner. Reasons for changing locations were named as relocation of or dissatisfaction with the first location.

### Burden related to MRI

40% (n = 42) of patients rated the MRI investigation as ‘not stressful at all’ while 3.9% (n = 4) ticked the highest level of stress. Top three stressors were noise (31.7%, n = 32), lying without movement (30.8%, n = 31) and narrowness (26.9%, n = 27). Fear of the scan-results was reported by 11.5%.

### Communication of MRI results

Out of 99 patients, 9% (n = 9) indicated that, to their knowledge, MRI images were compared to former scans. Both radiologists (51.5%, n = 51) and neurologists (44.4%, n = 44) performed these comparisons. 68% (n = 68) of the participants assessed the quality of physicians' delivery of MRI results as ‘elaborate’, 33% (n = 34) as ‘short’ and 2% (n = 2) had received no results at all. Nearly a quarter of the survey patients (23.7%, n = 23) at least once sought a second opinion on MRI results.

### Patient recommendations for an MRI education program

Concerning overall interest in an MRI education program, 61.5% (n = 64) marked ‘interesting’ and 28.8% (n = 30) even ‘extremely exciting’. Only 7.7% (n = 8) ticked ‘rather uninteresting’ while ‘not interesting’ was not mentioned.

**Figure 1:**
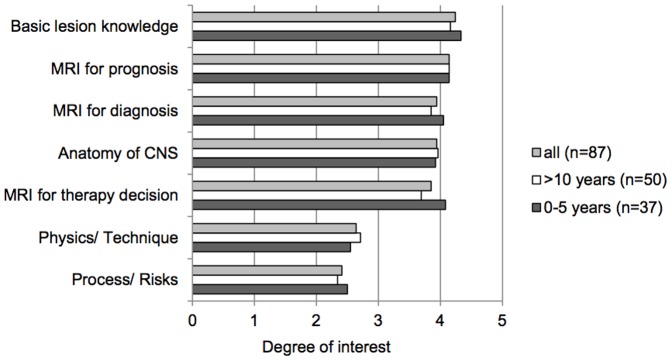
Degree of interest in MRI. Degree of interest is displayed with ratings from 0 ( = no interest) to 5( = high interest). Values are means. CNS =  Central nervous system.

For the possible content of a program, highest ratings were given for knowledge on different lesion types and their meaning (mean 4.24, SD 0.63 out of 5) and the value of MRI for the prognosis of MS (mean 4.14, SD 0.48 out of 5). Differences in interests between groups were minor, with the highest difference of 0.61 points in the area of treatment decisions based on MRI, which was considered more relevant in early patients (see figure 1).

When asked for a favorite presentation format, small group education programs not exceeding eight participants received the highest priority (58.6%), followed by brochures or leaflets (16.2%), individual trainings (14.1%), and online programs (8.1%). The majority of patients (51.5%) opted for a two to three hour training program, followed by a group session for one hour (20%). Only three patients voted for more than one session. Patients' goals for an MRI education program are given in table 3.

**Table 3:** Personal goals concerning MRI education (n = 99) (multiple answers possible).

	n	%
To achieve situational awareness	64	64.6
Better understanding of physicians	56	56.6
To develop own ideas	41	41.4
Shared decision making	38	38.4
Personal responsibility	34	34.3
To reduce anxiety about MRI investigation	7	7.1
To reduce anxiety about MRI results	7	7.1

Patients expressed hope for better understanding of their disease status through MRI knowledge. More than half of the participants thought that an MRI education can help them during communication with their physician, with one third hoping for more participation in decision making. In contrast, only few patients believed in a reduction of anxiety towards MRI results through MRI knowledge (6.7%, n = 7).

### MRI knowledge

MRI knowledge was fair and did not differ significantly between groups with a mean difference of 0.94 out of 17 points (early MS 10.51 points (SD 3.18), late MS 9.57 points (SD 3.39), p = 0.15, see table S2 in File S1). Subjective knowledge values (ranging from 0 to 10) were also comparable between groups: Patients with early MS estimated their MRI knowledge slightly lower with a mean of 4.27 points (SD 2.17) than patients with MS≥10 years with a mean of 4.49 points (SD 2.33). Objective MRI knowledge scores and subjective knowledge correlated significantly, but weakly with a Pearson correlation of 0.386 (p<0.05).

Basic anatomy questions to detect nose, cerebrum and spinal cord on MRI images were mostly answered correctly. Half of the participants (52.9%, n = 55) could name the lateral ventricles and 58.7% (n = 61) knew that computer tomography (CT) has a higher radiation exposure than the MRI. Only 25% (n = 26) were able to name the correct contrast agent used for MS patients (Gadolinium). 50% (n = 52) were aware of the limited information of a contrast-enhancing MRI shortly after a steroid pulse therapy. (For more details please see table S2 in File S1).

### Pilot MRI education program

The 90 minute power point based education program contains illustrative material, especially MRI images aiming to encourage participant involvement (for contents see table 4).

**Table 4:** Content of the MRI education program.

Principle of MRI technique
Risks and contraindications of MRI imaging.
The clinicoradiological paradox [20]
Rationale of gadolinium
Typical MS lesions and their evolution
Differentiation between new lesions and relapses
Typical locations of lesions and recent diagnostic MRI criteria
Anatomy of the CNS
MRI to measure treatment response [8]
Prognostic value for disability [5]

The program starts with a round of introductions, where all participants can state expectations and reasons for participating, and ends with a feedback round.

### Pilot evaluation study

The cohort consisted of 16 female and 10 male patients with a mean age of 46 years (SD 10 years). Most participants (69%, n = 72) had RRMS, with a mean disease duration of 6 years (SD 5 years). 16 (62%) participants had a higher education level and all had experienced at least one MRI with a mean number of MRI since disease onset of 8 (SD 6).

On a scale from 0 to 10, subjective knowledge increased from a mean of 3.71 (SD 2.01) before to a mean of 7.75 (SD 1.07) after the education program (p<0.001). Objective knowledge increased from 10.4 (SD 4.65) to a mean of 17.64 (SD 3.49) out of 24 possible points (p<0.001).

All patients emphasized the empowering effect of the program and overall agreement with the program's content was 3.22 out of a maximum of 4. The majority (92.3%, n = 24) was satisfied with program length and difficulty. 80.8% of participants (n = 21) completely agreed that the program should be recommended to other MS patients. 76.9% (n = 20) completely agreed that their knowledge on MRI has increased substantially and the remaining patients rather agreed. 92.3% (n = 24) of the participants felt capable of assessing the images at home after the training and felt that this knowledge would help them to cope with their disease.

All patients completely or rather agreed that the program would empower them to discuss their MRI results with their physicians. Patients did not express a need for more frequent MRI investigations, but 69.2% (n = 18) rather agreed to be able to co-decide on the usefulness of a future MRI investigation. A considerable number (38.5%, n = 10) would not trust to leave the diagnosing of their MRI images to their physician alone in the future. (For more details see table S3 in File S1).

## Discussion

MRI is of crucial relevance in diagnosing and managing MS. Although patients claim substantial MRI information needs [1], no study has yet addressed patients' attitudes, knowledge and detailed information needs concerning MRI in any detail. This study shows the vulnerable emotional situation especially of patients having their first MRI scan performed. They often feel they are at the mercy of a machine and the findings from the procedure. Although the process of giving information on MRI findings may differ between health care settings and countries, in most cases there will be a time lag between MRI performance and interpretation of the results, prolonging a phase of uncertainty, while patients might even have a report and/or a CD containing MRI scans at hand. Interviews indicated that this process requires better structuring. While interviewees explained substantial fear towards MRI results, survey results show that most patients were aware that lesions neither strongly correlate to disability nor to prognosis. This disagreement might be explained by the gap between somehow obtained general information about MRI and concrete findings in an individual case. One might assume that broad information could help to alleviate the stress elicited by MRI findings. However, interviews show that the timing for such information should not be too close to the diagnostic disclosure.

Interestingly, most patients thought that better MRI knowledge would help them to more actively participate within physicians' encounters. Knowing that more than 2/3 of patients claim active roles in encounters [14], MRI education might therefore enable more shared decision making. Even after an intervention as short as 90 min, 69% of the participants claimed that from now they would aim to assess their own images.

Answering an average of 10 out of 17 knowledge questions correctly, patients do possess a basic knowledge on MRI that can be built upon in an education program. Interestingly, knowledge on some basic aspects, such as radiation exposure and applicability of contrast agents, could only be answered by a minority of patients. Here, education might help to avoid unnecessary imaging soon after steroid treatment.

Beyond the expected knowledge increase directly after the short educational intervention, the substantial subjective knowledge increase together with the increased trust of patients to engage in physician encounters indicate the patient empowerment potential of the intervention. Complementary to the concept of shared-decision making [21], empowerment stresses more autonomy [22]. Interestingly, after the training, about one third does not want to leave the interpretation of MRI images to their physician. Eventually, participants tend to be skeptical towards results and interpretations of physicians, potentially causing distrust. This may indicate the need for further discussions about the challenging aspects of ‘expert patients’ [21].

As a limitation the response rate was low and based on a university outpatient cohort. Therefore findings might be biased towards higher educated, more interested patients. This means that knowledge might be even worse in less active patients, which emphasizes our findings. However, we cannot rule out that a substantial group of MS patients might not be interested in MRI education. Further work should look at consecutive patients in different treatment settings to overcome these limitations. As we did not obtain education level data, we cannot conclude on the actual impact on knowledge. We did not study possibly different views on MRI of females and males which also needs to be investigated in further work together with correlating individual MRI burden and perception of MRI.

In conclusion, this pilot work strongly supports further development of an evidence-based patient education program on MRI for patients with MS. However, our data already indicate that physicians should tailor their MRI communication strategies more to patient's preferences. These may substantially differ from the early diagnostic workup image to a follow-up scan during immunotherapy. Physicians need to be aware that a relevant amount of patients would even be happy to be able to read their own images to some extent. A controlled trial should be performed to show the added value to standard care as well as also possible side-effects. As patients' needs might substantially differ within the diagnostic process and the later disease course, these differences need to be studied in further work. Such a program should be developed and evaluated following the MRC's framework for the development and evaluation of complex interventions [23]. This pilot work offers an important preparatory basis for such a trial. Our data indicate that such an intervention might not only lead to more participation and empowerment, but also to a more rational use of health-care resources. This adds to previous studies, which have shown less demand of physicians and steroid treatments after relapse education [24] and a trend for increased adherence after thorough information on diagnosis, prognosis and early treatment effects for patients with early MS [25].

## Supporting Information

File S1 **Table S1, Systematic search in Pubmed (date of search Jan 5^th^, 2014). Table S2, Results of MRI knowledge questionnaire.** Given are mean numbers of correct answers and percentages in brackets. **Table S3, Evaluation of the education program: Statements and degree of consent.** *Level of agreement: 1 =  lowest level of agreement…4 = highest level of agreement. **Converted scores. **Survey S1, Qualitative Research: Item and questions of semi-structured interviews. Survey S2, MRI knowledge questionnaire.**
(DOCX)
