# Supplementary material for: Magnetic Resonance Imaging in Multiple Sclerosis – Patients' Experiences, Information Interests and Responses to an Education Programme
Source: PLoS One. 2014 Nov 21;9(11):e113252. doi: 10.1371/journal.pone.0113252 (PMC4240649; doi:10.1371/journal.pone.0113252)
Supplement: File S1 — Table S1, Systematic search in Pubmed (date of search Jan 5th, 2014). Table S2, Results of MRI knowledge questionnaire. Given are mean numbers of correct answers and percentages in brackets. Table S3, Evaluation of the education program: Statements and degree of consent. *Level of agreement: 1 = lowest level of agreement…4 = highest level of agreement. **Converted scores. Survey S1, Qualitative Research: Item and questions of semi-structured interviews. Survey S2, MRI knowledge questionnaire. (DOCX) [file pone.0113252.s001.docx]

**Supplemental File S1**

**Table S1**

**Systematic search in Pubmed (date of search Jan 5^th^, 2014)**

|  | **Search terms** | **hits (n)** |
| --- | --- | --- |
| #4 | #1 AND #2 AND #3 | **312** |
| #3 | "Magnetic Resonance Imaging"[Mesh] OR magnetic resonance imaging OR mri | 404470 |
| #2 | "Multiple Sclerosis"[Mesh] OR "Myelitis, Transverse"[Mesh] OR "Demyelinating Diseases"[Mesh] OR "Encephalomyelitis, Acute Disseminated"[Mesh] OR (("multiple sclerosis" OR "transverse myelitis" OR "optic neuritis" OR “adem” OR "neuromyelitis") | 87635 |
| #1 | education OR "patient education" OR "education* method*" OR "education* material*" OR "education* program*" OR (information AND coping) OR "patient information*" OR "health information*" OR "information* method*" OR leaflet* OR lecture* OR "communications media" OR "information sheet*" OR "patient guidance" OR brochure* OR pamphlet* OR counselling OR "patient counselling" OR "telephone call*" OR "web site*" OR website* OR (teaching AND computer*) OR (audiovisual AND information) OR "decision making" OR "shared decision making" OR "informed choice" OR "decision support" OR advice OR "Health Education" OR "Consumer Health Information" OR "Decision Making" OR "Decision Support Techniques" OR "Informed Consent" OR "Communication" OR "Patient Participation" OR "Self Care" OR "Health Status Indicator*" OR "Drug Information Services" OR "Information Dissemination" OR "Access to Information" | 1431925 |

**Survey S1**

**Qualitative Research: Item and questions of semi-structured interviews**

Gender:

Age:

Diagnosis of MS (year):

Disease Course:

Treatment:

MRI experience (first and most recent, frequency):

What do you feel when looking at the image (transversal scan with eyes cut) below?

How was your first time in the MRI scanner? Can you describe it?

Do you remember the first time looking at your own MR images? How did it take place?

Are you interested in participating in an MRI education program? Why?

How much time would you be able or willing to spend?

In which manner would you like to have the information presented?

Please rank the following topics. How much are you interested in them if 0 means no interest and 5 means highest interest?

- Basics of the MRI (contrast agent, physics)
- The process of an MRI scan
- Neuroanatomy and MS symptoms
- Lesions and their meaning
- MRI used to diagnose MS
- MRI used to make a prognosis of the disease course
- MRI to measure the effectiveness of a treatment

Do you feel frightened or do you have any concerns towards an MRI education program?

How much do you already know about MRI?

Can you show me the MS lesions on the image (example T2 scan)?

**Survey S2**

**MRI knowledge questionnaire**

1) Please relate the numbers in the image to the correct anatomical structures.


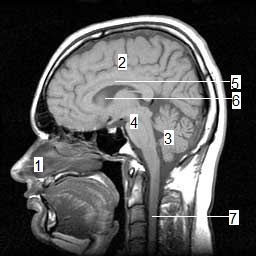


1) Nose

2) Cerebrum

3) Cerebellum

4) Brain stem

5) Corpus callosum

6) Ventricle

7) Spinal cord

2) How much is the radiation exposure of magnetic resonance imaging (MRI) compared to the radiation exposure of computer tomography (CT)?

- Same radiation exposure in CT and MRI.
- No radiation exposure in CT and high radiation exposure in MRI.
- No radiation exposure in MRI and high radiation exposure in CT.
- Both investigations do not cause any radiation.

3) Which contrast agent is often used for multiple sclerosis patients?

- Barium sulfate
- Gadolinium
- Iodine-containing contrast agent
- Carbon dioxide

4) In which sequence is the contrast agent visible?

- In T1.
- In T2.

5) How many lesions can you find in the image on
the right?


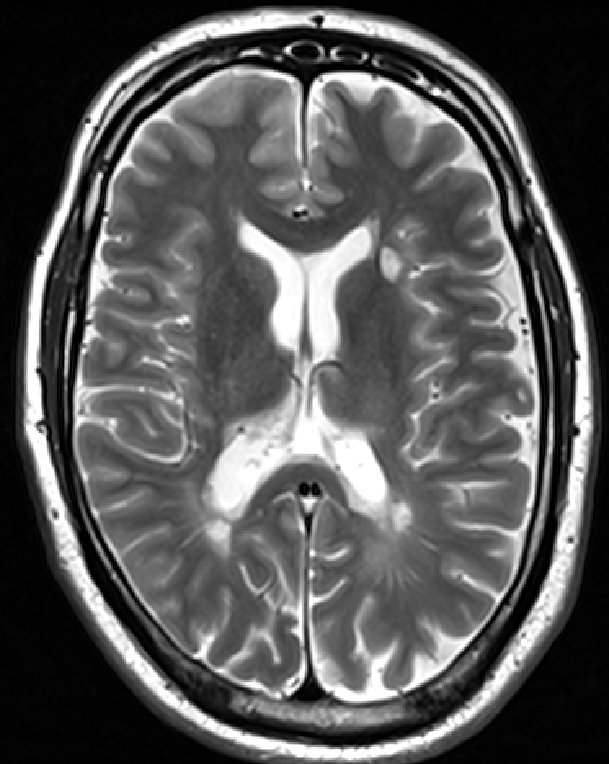


- One
- Two
- Three
- More than five.

6) What does ‚black hole‘ mean?

- An abscess-like accumulation of fluid.
- An area, where neuronal tissue is irreparably destroyed.
- A sign of reconstruction of the myelin sheath.
- An active lesion, where the immune system is attacking nerve cells.

7) Is it possible to estimate the course of MS reliably from the number of lesions on an MR image?

- Yes.
- No.

8) Is it possible to estimate the degree of disability from an MR image?

- Yes.
- No.

9) Does a large number of contrast enhancing lesions on the image anticipates a relapse?

- Yes.
- No.

10) Is it reasonable to do an MRI investigation with contrast agent shortly after a cortisone pulse therapy?

- Yes.
- No.

11) Which of the following statements is not correct?

- MRI is useful in the early stage of the disease.
- As a principle, multiple sclerosis patients should have MRI once a year.
- Regular MRI investigations are helpful during immunotherapy.
- Besides diagnosis MRI is especially useful to monitor treatment effects.

Results:

Early MS (0-5 years disease duration): 10.51 out of 17 points (SD 3.18)

Late MS (>10 years disease duration): 9.57 out of 17 points (SD 3.39)

All: 9.96 out of 17 points (SD 3.32)

| **Table S2:** Results of MRI Quiz | |  |  |
| --- | --- | --- | --- |
| Topic | Group 1  (0-5 years) | Group 2  (>10 years) | all |
| See survey S2 for details | (n=43) | (n=61) | (n=104) |
| 1. Anatomy-nose | 41 (95.3) | 57 (93.4) | 98 (94.2) |
| 2. Anatomy-cerebrum | 40 (93.0) | 51 (83.6) | 91 (87.5) |
| 3. Anatomy-cerebellum | 32 (74.4) | 36 (59.0) | 68 (65.4) |
| 4. Anatomy-spinal cord | 41 (95.3) | 47 (77.0) | 88 (84.6) |
| 5. Anatomy-brain stem | 28 (65.1) | 27 (44.3) | 55 (52.9) |
| 6. Anatomy-corpus callosum | 20 (46.5) | 21 (34.4) | 41 (39.4) |
| 7. Anatomy-ventricle | 25 (58.1) | 30 (49.2) | 55 (52.9) |
| 8. Radiation exposure | 24 (55.8) | 37 (60.7) | 61 (58.7) |
| 9. Contrast agent | 12 (27.9) | 14 (23.0) | 26 (25.0) |
| 10. Sequences T1, T2 | 8 (18.6) | 10 (16.4) | 18 (17.3) |
| 11. Lesion count | 29 (67.4) | 39 (63.9) | 68 (65.4) |
| 12. Black hole | 19 (44.1) | 27 (44.3) | 46 (44.2) |
| 13. Value of MRI-disease course | 38 (88.4) | 51 (83.6) | 89 (85.6) |
| 14. Value of MRI-disability | 30 (69.7) | 36 (59.0) | 66 (63.5) |
| 15. Value of MRI-relapse predict | 27 (62.8) | 44 (72.1) | 71 (68.3) |
| 16. MRI and steroid therapy | 21 (48.8) | 31 (50.8) | 52 (50.0) |
| 17. Frequency of investigations | 17 (39.5) | 26 (42.6) | 43 (41.3) |

Given are mean numbers of correct answers and percentages in brackets.

**Table S3:** Evaluation of the education program: Statements and degree of consent

| Evaluation of the education program | Level of agreement* |
| --- | --- |
| Part 1: Satisfaction with the program | Sub-score**: 3.22 |
| 1.1 Altogether, I am satisfied with the education program. | 3.96 |
| 1.2 I was interested in the contents. | 3.62 |
| 1.3 The program was too extensive/ long. | 1.62** |
| 1.4 There was a good balance between training and breaks. | 3.36 |
| 1.5 The program was too difficult. | 1.52** |
| 1.6 To many technical terms were used. | 1.96** |
| 1.7 I was able to clarify my questions and issues. | 3.54 |
| 1.8 There was sufficient participation of the group. | 3.73 |
| 1.9 I would recommend the program to MS-patients. | 3.85 |
| Part 2: Perceived effects | Sub-score**: 3.22 |
| 2.1 My knowledge on MRI has increased significantly. | 3.77 |
| 2.2 I learned how to rate my MRI results. | 3.46 |
| 2.3 I can now understand the significance of my MRI results for the overall assessment of my disease. | 3.58 |
| 2.4 I have more control about my disease now. | 2.65 |
| 2.5 I lost fear of the MRI investigation. | 2.87 |
| 2.6 I lost fear of the MRI results. | 2.92 |
| 2.7 The imparted knowledge helps me to cope with my disease. | 3.31 |
| Part 3: Conversion into action | Sub-score**: 3.59 |
| 3.1 Now, I can discuss MRI results with my physician. | 3.36 |
| 3.2 In the next medical consultation I would like to talk more about the MRI. | 3.48 |
| 3.2 Now, I feel more competent in an encounter with my physician. | 3.32 |
| 3.3 I am now able to co-decide whether an MRI would be useful. | 3.23 |
| 3.4 I would like to have more frequent investigations now. | 1.83** |
| 3.5 I am now able to take a look at my MRI images at home. | 3.23 |
| 3.6 Despite of my new knowledge, I leave the diagnosing to the doctors. | 2.73 |
| * Level of agreement: 1=lowest level of agreement…4=highest level of agreement | |
| ** converted scores. | |
